# Policy and programmatic considerations for introducing a longer-acting injectable contraceptive: perspectives of stakeholders from Kenya and Rwanda

**DOI:** 10.9745/GHSP-D-14-00106

**Published:** 2014-12-02

**Authors:** Kevin McKenna, Jennet Arcara, Kate H Rademacher, Caroline Mackenzie, Fidele Ngabo, Emmanuel Munyambanza, Jennifer Wesson, Elizabeth E Tolley

**Affiliations:** a FHI 360, Durham, NC, USA; b University of North Carolina at Chapel Hill, Chapel Hill, NC, USA; c FHI 360, Nairobi, Kenya. Now with Ipsos, Nairobi, Kenya; d Ministry of Health, Kigali, Rwanda; e FHI 360, Kigali, Rwanda. Now with Company for Research in Social, Behavior and Health (RSH) Ltd, Kigali, Rwanda; f FHI 360, Kigali, Rwanda. Now with IntraHealth International, Chapel Hill, NC, USA

## Abstract

**Background:**

More than 40 million women use injectable contraceptives to prevent pregnancy, and most current or previous injectable users report being satisfied with the method. However, while women may find injectables *acceptable*, they may not always find them *accessible* due to stock-outs and difficulties with returning to the clinic for reinjections. FHI 360 is spearheading efforts to develop a longer-acting injectable (LAI) contraceptive that could provide at least 6 months of protection against pregnancy. This article addresses systems-level considerations for the introduction of a new LAI.

**Methods:**

We conducted qualitative case studies in Kenya and Rwanda—two countries that have high levels of injectable use but with different service delivery contexts. Between June and September 2012, we conducted in-depth interviews with 27 service providers and 19 policy makers and program implementers focusing on 4 themes: systems-level barriers and facilitators to delivering LAI services; process for introducing an LAI; LAI distribution approaches; and potential LAI characteristics. We also obtained electronic feedback from 28 international family planning opinion leaders.

**Results:**

Respondents indicated strong interest in an LAI and thought it would appeal to existing injectable users as well as new family planning clients, both for spacing and for limiting births. Providers appreciated the potential for a lighter workload due to fewer follow-up visits, but they were concerned that fewer visits would also decrease their ability to help women manage side effects. The providers also appreciated the 1-month grace period for follow-up LAI injections; some seemed unaware of the latest international guidance that had increased the grace period from 2 weeks to 4 weeks for the currently available 3-month injectable. The majority of policy makers and program implementers were supportive of letting community health workers provide the method, but many nurses and midwives in Kenya had reservations about the approach. At the policy level, respondents indicated that obtaining regulatory approvals before introducing the new method could be costly and time-consuming. Manufacturing and procurement decisions could also affect cost and availability.

**Conclusions:**

Successful introduction of a potential longer-acting injectable may be enhanced by considering broader systemic issues, including managing cost to the health system and users, expanding access through community-based distribution, and training providers on the latest service delivery guidelines.

## Abstract (precis)

Unique attributes of a longer-acting injectable would likely appeal to both existing injectable users and new clients, both for spacing and for limiting births, and allow health systems to operate more efficiently. Considerations for enhancing successful introduction of this potential new method include keeping the cost low, expanding access through community-based distribution, and training providers to improve practices about injectables in general.

## BACKGROUND

Between 1995 and 2005, use of injectable contraceptives more than doubled worldwide, from 12 million married women to over 32 million.^1^ Currently, more than 40 million women use injectables to prevent pregnancy.^2^ In studies of current and previous injectable users, more than three-quarters of women report being satisfied with the method, mainly due to its inherent product characteristics: It requires relatively few visits to a health care facility, provides discreet contraceptive protection, is less user-dependent than methods such as condoms or oral contraceptive pills, and requires less invasive medical procedures than methods such as intrauterine devices (IUDs) or implants.^3^^–^^7^

While women may find the injectable *acceptable*, they may not always find it *accessible*. Multiple factors ultimately affect clients' ability to obtain and continue using contraceptive methods, including policy and service delivery guidelines, recommendations and decisions about appropriate target populations, pricing, and product distribution mechanisms. Logistical issues at the systems, facility, and individual levels may also impede access and continuation, causing clients to unintentionally disrupt or discontinue contraceptive adherence. Specifically, commodity stock-outs at facilities can be common, and clients may also have difficulty returning to the clinic for reinjections. Similarly, when women return late for their follow-up appointments, they are sometimes denied a reinjection, even when they are within the approved grace period.^3^^–^^5^^,^^8^^–^^13^

Commodity stock-outs and difficulty returning to the clinic for reinjections can impede access to injectables.

A project led by FHI 360 with funding from the Bill & Melinda Gates Foundation is underway to develop an innovative injectable contraceptive that could provide at least 6 months of protection from pregnancy. Building on the popularity of existing 1- and 3-month injectables, a longer-acting injectable (LAI) could address some of the access-related barriers that contribute to discontinuation and could provide women with greater contraceptive choice.

Development of a longer-acting injectable that could provide at least 6 months of pregnancy protection is currently underway.

During the first phase of the LAI development initiative, we used qualitative methods in Kenya and Rwanda to explore acceptability, potential demand, and policy and delivery issues related to a new LAI. Kenya and Rwanda were selected because injectables are by far the most popular family planning method in both countries.^8^^,^^14^ Despite widespread acceptability and use, several contextual factors differ between the two countries, providing useful diversity in a case-study approach. For example, the method mix differs with a higher percentage of women in Kenya than in Rwanda using long-acting methods. In addition, Rwanda has a large public-sector distribution network and well-established community-based distribution (CBD) programs that cover the entire country. In contrast, Kenya has complementary public and private markets, and while a CBD system for administering the DMPA injectable has been piloted and permitted through recent policy change, it has yet to be fully integrated into current national programs.^7^^,^^8^

In this article, we identify potential barriers and opportunities related to the introduction of an LAI at the policy, health system, clinic, and client levels. A companion paper published in *Global Health: Science and Practice* described the preferences of opinion leaders, providers, and potential users for a range of potential characteristics of an LAI.^15^

## METHODS

Between June and September 2012, we conducted in-depth interviews (IDIs) with 19 policy makers and program implementers and 27 service providers in Kenya and Rwanda (Table 1). In addition to nurses and midwives from mostly urban areas, service providers included community health workers (CHWs) recruited from private- and public-sector clinics in peri-urban and rural areas who provide injectables. Policy makers and program implementers represented local and international government and private/nongovernmental family planning programs at a variety of levels. Some of the policy makers worked at nongovernmental organizations (NGOs) as policy analysts or on developing family planning and other reproductive health policies with their colleagues from government. Program implementers included NGO directors and senior program officers, government program directors, and program managers.

**Table 1.** Profile of In-Depth Interview Respondents from Kenya and Rwanda (N = 46)

****	**Kenya**		**Rwanda**		**Totals**
**Role in the Health System**	**Public Sector**	**NGO/Private Sector**	**Public Sector**	**NGO/Private Sector**	
Program implementers	2	5	1	2	10
Policy makers	2	3	2	2	9
Service providers					
Nurses/midwives					
Rural	–	–	1	–	1
Peri-urban	7	–	2	–	9
Urban	2	3	1	2	8
CHWs					
Rural	–	–	6	–	6
Peri-urban	3	–	–	–	3
Urban	–	–	–	–	–
**Totals**	**16**	**11**	**13**	**6**	**46**

Abbreviations: CHWs, community health workers; NGO, nongovernmental organization.

The IDIs followed a guide that focused on 4 themes:

1. Systems-level barriers and facilitators to contraceptive delivery services in general and as related to a possible LAI
2. Steps needed to introduce an LAI into existing programs and facilities
3. Possible new LAI distribution approaches
4. Exploration of LAI characteristics identified in the target product profile (TPP)

The TPP is used to inform product development and identifies both desired and minimally acceptable targets related to effectiveness, the target user population, side effect profile, dosage and delivery mechanism, cost, and other aspects (Box 1). LAI characteristics from the TPP were emphasized with all interviewees.

**BOX 1. Target Product Profile for a Longer-Acting Injectable Contraceptive**

Goals for this new method:

- 99% effective in preventing pregnancy when used correctly
- Indicated for women of all reproductive ages*
- No contraindications—can be used immediately after birth and does not interfere with breastfeeding*
- Return to fertility when stopping the method similar to that among women who have stopped using a nonhormonal contraceptive method
- New dose every 6 months with a 1-month grace period*
- Given in the arm
- Side effects no worse than those associated with currently available hormonal methods/injectables*
- Can be stored in warm, humid climates*
- Costs US$4 or less per year in public-sector programs*
- Single-dose, prepackaged, disposable injection system
- Can be provided by trained community health workers*

* Characteristics addressed in this article.

IDIs were conducted by male or female interviewers in English, French, or the local language (as preferred by the participant). Interviewers used illustrations depicting each TPP characteristic to facilitate discussion. IDIs were audio-recorded and then translated into French or English and transcribed. The documents were then uploaded into NVivo9, and the information was coded by multiple coders and reviewed for reliability. To analyze the data thematically, we wrote detailed memos describing subthemes related to each main code, including each TPP characteristic. We also created Excel matrices to examine variations in subthemes by country and participant type. The study was approved by FHI 360's Protection of Human Subjects Committee and by the institutional review boards in both countries.

In addition, reproductive health specialists from FHI 360 identified 67 international opinion leaders with expertise in family planning for an open-ended email-based survey about the perceived need and important characteristics for an LAI as well as potential challenges related to LAI development and introduction. Respondents were asked to identify additional opinion leaders and to provide their contact information; 28 individuals were identified through this mechanism, for a total of 95. Of the 95 whom we contacted, 28 responded (Table 2). Survey responses were organized into a matrix by topic.

**Table 2.** Profile of International Opinion Leaders Responding to Email-Based Survey (N = 28)

**Characteristics**	**No. of Respondents**
Type of Organization	
NGO (international and based within the US)	14
International organization (UN, etc)	3
University	4
Donor	5
Government	1
Clinical services	1
Countries	
Global (work in > 1 country)	18
Brazil	1
Guinea	1
Jordan	2
Malawi	1
Nigeria	1
Uganda	2
United States	2

## RESULTS

Below, we discuss stakeholder perspectives in 4 main areas: (1) health systems-level considerations for LAI introduction; (2) opportunities and barriers for introduction at the clinic level; (3) distribution mechanisms to ensure wide access to the method; and (4) identification of potential LAI users.

### Health Systems-Level Considerations

When asked about the decision-making and planning processes required to introduce a new contraceptive product, respondents noted that regulatory processes, manufacturing and procurement, and cost were the main considerations that could affect access to and eventual uptake of a new LAI.

#### Regulatory Policy Approvals

Six of the 28 opinion leaders responding to the email-based survey mentioned the need to obtain regulatory approvals before introducing a new contraceptive, with several pointing out that the process is often lengthy and costly. Similarly, 6 of 9 policy makers in Kenya and Rwanda stated that potential users' access to new drugs is significantly affected by the series of international and country regulatory approvals (eg, prequalification from the World Health Organization [WHO], addition to international and country-level Essential Medicines Lists). Additionally, 6 policy makers and program implementers in the two countries indicated such a product would first need a small-scale pilot introduction to assess programmatic feasibility, safety, and acceptability, which could pose a potential obstacle to swift introduction.

#### Supply Chain Management: Manufacturing, Procurement, and Associated Costs

Logistics involving the supply chain, specifically manufacturing, procurement, and associated costs, were concerns for a number of the IDI and survey respondents. Several Kenyan and Rwandan program implementers explained that their country's reliance on international-level decisions for procuring contraceptive commodities commonly led to delays in their arrival in-country. Six opinion leaders suggested that the price (and subsequently, cost to users) of a new LAI could be reduced if it were manufactured in a country with low labor costs and proximal shipping distances. One opinion leader from a global NGO said that product developers “should target low-cost manufacturing to keep costs low [and should] not license to [a] high-cost manufacturer,” and another was adamant that the rights to an LAI “should rest in ‘public hands’ such that several manufacturers can produce it without hindrance.”

Partnering with a manufacturer with low labor costs and proximal shipping distances could help keep the cost of a new longer-acting injectable low.

Three Kenyan policy makers and program implementers noted that current procurement processes were sometimes lengthy and could potentially delay any future introduction efforts for an LAI; similarly, program implementers and policy makers in both countries noted the numerous steps and governing bodies involved in moving from initial product introduction to making the method available to women in the country. The country director for an NGO in Kenya stated:

That's the problem; we don't have a local manufacturer … we're not like India where they're manufacturing almost all their family planning commodities so they can at least deliver it quickly to where it's needed.

Similarly, 8 service providers in Kenya suggested that the shelf life of any LAI should be at least 3 years, with some specifying that this would enable the product to withstand the lengthy overseas procurement and local distribution processes.

#### Funding Contraceptive Commodities

One-third of Kenyan policy makers and program implementers and one-half of the Rwandan counterparts pointed out that the cost of an LAI would largely determine accessibility. They explained that most contraceptives are procured using government or donor funds, which could translate to limited stocks in the event of a shortage of donor funds or Ministry of Health (MOH) allocations. Twelve opinion leaders mentioned in their open-ended responses that an LAI should be affordable to procurement agencies, with a price point similar to or less than currently available injectable products. Additionally, a few respondents in Kenya and Rwanda mentioned that decisions about what contraceptives to include in the national method mix is a process driven by available resources, taking into account funding, cost, and the variety of methods available; therefore, introducing a new method might involve reducing financial allocations for other existing methods.

Many of the respondents in Kenya and Rwanda thought cost would be the determining factor in accessibility to a new longer-acting injectable.

Interestingly, this differs somewhat from the user perspective as reported more fully in the companion paper by Tolley and colleagues,^15^ wherein potential users ranked cost as one of the least important issues. Potential users' perspectives on cost were influenced by whether their country provides contraceptives for free in the public sector; potential users in Kenya, where paying for contraceptives through social marketing or the private sector is relatively common, were more willing to pay for an LAI than those in Rwanda.

### Clinic-Level Barriers and Opportunities

Some LAI characteristics could affect service provision at the clinic level, which would, in turn, affect potential users' access to an LAI. The TPP used in the interviews specified a dosing schedule of 2 reinjections per year; a grace period, or reinjection window, of 1 month; and that the LAI should not require cold chain transportation and storage.

Overall, providers felt an LAI would fit into existing programs well, both because they thought that clients would be interested in the method type and duration and because they perceived that certain product characteristics could help relieve them of job-related stress. While providers did acknowledge that there may be initial difficulty or learning curves in time management, clinic workflow, and side effect management, they remained mostly positive about the potential of an LAI to enhance both client and provider satisfaction.

#### Dosing Schedule and Follow-Up Appointments

According to WHO guidance, the grace period for follow-up injections for the currently available 3-month injectable is 4 weeks, during which time women do not require additional contraceptive protection.^16^ Before WHO updated this guidance in 2008, the approved grace period was 2 weeks. In Kenya and Rwanda, some providers seemed unaware of this updated guidance because they thought the 1-month grace period for the LAI TPP was significantly different from the current reinjection window.

Most service providers in Kenya (12 of 15) agreed that a 6-month duration for the method would be desirable, while an additional few added that effectiveness up to 1 year would also be desirable. In Rwanda, 4 providers mentioned that everyone—women, providers, facilities—would benefit from a dosing schedule with fewer follow-up appointments.

Providers described a variety of ways in which they manage reinjection windows for injectables. While some providers in the two countries allow women the full grace period in which to return for reinjection, other providers intentionally advise women that the grace period is much shorter than what it really is or do not mention a grace period at all to ensure that women return on time. The majority of respondents in both countries thought that 4 weeks would give women sufficient flexibility to get to a facility or CHW for LAI reinjections. In Rwanda, 10 providers reported that they have problems with women returning late for follow-up injections, and 7 providers felt that a 4-week grace period would enable them to maintain continuation for more women. One CHW explained:

It [the seemingly longer grace period] is really good because [currently] if we count 2 weeks that a mother hasn't respected her appointment, we can't give her the method. If now it's a month, we are lucky, we will be sure that the service that we provide is impeccable.

In Kenya, 4 policy makers and 3 program implementers were concerned that discontinuation rates might be higher for an LAI (compared with currently available contraceptives) if side effects could not be managed properly as a result of longer periods between clinic visits. A Kenyan provider worried that clients would suffer side effects for longer periods of time without obtaining assistance if the reinjection schedule did not require clients to return as frequently. One provider worried about irreversibility in the event of severe side effects:

Some respondents worried that longer periods between clinic visits for reinjections would make it difficult to manage side effects properly.

My concern would be if I inject the client and then we have this bleeding or somebody develops maybe blood pressure and maybe the drug will take a year or maybe 2 years [respondent's understanding of the potential duration of effectiveness], what will happen in between?

Several providers in Kenya—and several potential users themselves (see Tolley et al, 2014^15^)—noted that the irreversibility of an LAI could be problematic, either in relation to side effect management or if a woman changed her fertility intentions; both providers and users suggested that a way to reverse the drug could be important and useful. Conversely in Rwanda, only 1 respondent, a service provider, thought that side effects would be more difficult to manage with a lengthened dosing schedule.

#### Workload and Learning Curve for Providers

In both countries, most respondents thought that providers' workload would decrease with introduction of an LAI because of longer intervals between injections. A provider from Rwanda remarked favorably that workload is sometimes substantial but would lessen with the introduction of an LAI:

Work will decrease because the frequency of clients will also decrease … we will do our work better because sometimes it happens that we don't do our work like we should because of the pressure of a line of people waiting outside the door. But when they are fewer, you can put your things in order without problems.

Six opinion leaders agreed that an LAI would reduce the burden on the health care system because of longer intervals between reinjections.

Most respondents agreed that longer intervals between injections would reduce the burden on the health care system.

However, several service providers from both countries mentioned that their workload might initially increase due to a perceived steep learning curve, as users would need to be educated about the method, many women would want to try it, and increased monitoring paperwork might be needed for a new method. As policy makers and program implementers pointed out, the high client volume many providers confront already favors administration of injectables over longer-acting methods, since from the providers' perspective, it is generally faster and simpler to give a woman an injection than to perform the more involved procedures required of IUDs and implants.

#### Storage and Stock-Outs

All respondents in both countries agreed that an LAI with no cold chain storage requirements was essential to ensure that a product could feasibly reach more users. In an open-ended question about important product characteristics, 9 opinion leaders also raised this as a priority. In Rwanda, all service providers volunteered that the ability to store an LAI without refrigeration would ensure wide access to the method. A CHW from Rwanda remarked:

What's really good is that we, community health workers, we don't have refrigeration … so for us, a good medicine is one that we can use without difficulty and one in which we can have confidence in how it is stored. If you give CHWs medicines that require a fridge, it's expected that there would be many [commodity] losses.

Respondents acknowledged that stock-outs can limit access for potential users by delaying the initiation or continuation of a method, with stop-gap measures (such as condoms) providing less effective pregnancy prevention. Although not related directly to a TPP characteristic, several Kenyan and Rwandan providers reported that good record-keeping and planning help to avoid stock-outs at the clinic level and could also be applied to an LAI to ensure consistent access.

### Distribution Mechanisms to Enhance Access

The TPP of the LAI included a goal of product distribution by CHWs. Respondents had differing levels of experience with CBD of family planning methods; their opinions about CBD of an LAI tended to be based on provider type and their familiarity with CBD programs. Respondents were divided about the possibility of self-injection of an LAI.

#### Community-Based Distribution

CBD of injectables has been ongoing in Rwanda since 2010, with a specific focus on rural areas, and most Rwandan respondents were positive about the program. With the exception of 3 providers, Rwandan respondents felt that a properly trained CHW should have a role in providing an LAI, especially for rural women. One nurse at a rural health center noted:

It's also an advantage for the women who live far from health centers, and also it helps approach more people and sensitize them [to family planning] since we don't do field visits.

A few Rwandan providers objected to CHWs providing an LAI, and they further expressed that CHWs did not have enough training to administer injections in general.

Although CBD of injectables is not widely available in Kenya, all but 2 policy makers and program implementers were very supportive of delivering an LAI through community-based mechanisms; they projected that task sharing with CHWs would save time both for clients and for facility staff and would better serve rural areas. Several policy makers and program implementers mentioned current pilots or earlier programs in which contraceptives were successfully provided at the community level. One government official stated:

It's not happening now, but it's likely to happen; there are efforts to scale it down to the community level by training the community health workers on the safety of injections and to provide those services in the hard-to-reach areas, and especially [places] where we're facing challenges of retaining or recruiting [staff from] medical training colleges.

However, only one-quarter of providers in Kenya envisioned this possibility. Common concerns about CBD of an LAI, or of any injectable, elucidated by nurses and midwives in Kenya (the largest provider group in the sample) included worries that CBD workers would not have adequate safe-injection training, proper resources to dispose of used needles, or the ability to manage side effects. One provider explained:

A community health worker may inject well, but when it comes to the biology … in case of any side effects that need explanation, a community health worker may not be in a position to assist appropriately.

Many nurses and midwives in Kenya worried that community health workers would not be able to provide injectables safely and effectively.

Among international opinion leaders, 6 mentioned that an LAI could ideally be administered by CHWs, with only 1 opinion leader expressing concern for potential confusion among CHWs when having to distinguish between the different types of injectables (ie, between the 3-month injectable and an LAI).

#### Self-Injection

With the introduction of Sayana Press, a DMPA product prepackaged in a single-use Uniject injection system administered subcutaneously, self-injection of injectable contraceptives may be feasible in the future. Respondents in both Kenya and Rwanda were divided on whether self-injection of an LAI would be a good option for women. In Rwanda, 5 respondents agreed that self-injection could work, with caveats; 6 respondents strongly disagreed; and 2 respondents were non-committal. In Kenya, 9 respondents were not supportive of the idea, while 4 were fully supportive, and 6 others pointed out both advantages and disadvantages to the approach. There was no clear pattern by job function in either site.

Providers who agreed with the idea saw the potential for reduced workload for providers and tended to relate self-injection of a contraceptive to self-injection of insulin by patients with diabetes; they reasoned that if people with diabetes could be trained to self-inject and could manage their fears about injection, so could contraceptive users:

That would be very interesting because it would decrease the work [at the facility]. People could follow their [family planning] program without coming to the hospital. If we trained them like we do for those who have diabetes …

Providers who disagreed foresaw problems with infection prevention, insecure storage of the product at women's homes, improper management of contraindications and side effects, and women's fear of the pain of injection as an inhibitor of timely and correct injections. A CHW, laughing in response to the question, explained:

Women are afraid of injections, they wouldn't dare do it themselves. And then they might keep it at their house without using it like they do sometimes with other medications. If there is no follow-up like we do, then all the women would surely get pregnant.

In open-ended email responses, 2 opinion leaders suggested that self-injection was an ideal characteristic for an LAI and specified a preference for a subcutaneous self-injectable via the Uniject delivery device.

### Perspectives About Potential LAI Users

A TPP goal is to develop an LAI that could be used by all women of reproductive age—regardless of age or parity and without any contraindications—but especially by women immediately after childbirth and by breastfeeding mothers.

In general, respondents in both countries thought that while a wide range of women would be interested in an LAI, current satisfied injectable users might be logical early adopters who might switch to an LAI because it would be similar to the 3-month injectable but more convenient.

Most Kenyan policy makers and program implementers, along with 6 providers, stated that women who wanted to space their children would be especially interested in an LAI. However, Rwandan respondents envisioned an LAI to be used more for limiting births than for spacing births, and especially among women who have less familiarity and comfort with other longer-acting methods commonly used for limiting such as IUDs and implants.

Several respondents in both countries mentioned that an LAI would be advantageous because it would fill what they saw as a gap between currently available short-acting and long-acting methods. The potential for cost savings to women and couples if a 6-month product were priced approximately the same as a 3-month product was also noted by some respondents.

Several respondents thought a longer-acting injectable would fill a gap between currently available short-acting and long-acting methods.

Respondents also acknowledged that certain subpopulations of potential users might be particularly interested in or appropriate for an LAI. In both countries, almost all service providers and several country-level stakeholders saw the need for an injectable product that could be used immediately after childbirth. Respondents differed a bit on their attitudes toward the use of an LAI by adolescent girls. Respondents in both countries acknowledged that school-aged girls are already interested in injectables but are often not having their contraceptive needs met for a number of reasons. There was some concern from Kenyan respondents about parental consent for minors or the potential for young women using the injectable to disregard using condoms to protect against sexually transmitted infections. In Rwanda, respondents were particularly supportive of adolescent use of an LAI with no contraindications; for some service providers, their support was driven by an inaccurate belief that the 3-month injectable is contraindicated for young women because of its perceived potential for sterility in women who do not have proof of fertility through previous pregnancy.

Few providers in either country were well-versed about whether the pharmaceutical formulation of hormonal contraception could increase susceptibility to and transmission of HIV infection, although policy makers and program implementers were familiar with recent WHO guidance and MOH statements released on the matter. Also, when asked about HIV, some respondents in both countries acknowledged the lack of HIV prevention inherent to a non-barrier method. Some respondents also understood questions about HIV and hormonal contraception as whether an LAI could be used by women with HIV. Their responses reflected their opinions about whether women with HIV should have children, or they mentioned the potential interaction between hormonal methods and drugs typically taken by women with HIV (eg, antiretrovirals, tuberculosis treatment).

Policy maker, program implementer, and provider perspectives on overall interest in and acceptability of the product, as well as appropriate target user types, were generally consistent with potential users' interest as presented in the companion paper by Tolley et al.^15^ Generally, both potential users and other respondents ranked effectiveness as a very important characteristic for a potential LAI, prioritized a predictable return to fertility, and preferred a delivery system with a single, prepackaged disposable injection. Potential users were actually less concerned about side effects than were providers, perhaps reflecting providers' anticipation or worry that side effects of an LAI would require more of their time or would be more difficult to manage.

## DISCUSSION

Both international and country-level stakeholders play a pivotal role in shaping access to and demand for new contraceptive products. A representative sample of such key stakeholders identified a number of possible barriers to and opportunities for increasing access to an LAI at the policy, facility, distribution, and user population levels. Using the access “architecture” proposed by Frost and Reich,^17^ their insights are organized into 3 distinct components (affordability, availability, and adoption) that, when taken together, comprise the conceptual model showing foundation for access to an LAI (Figure).

**Figure.**
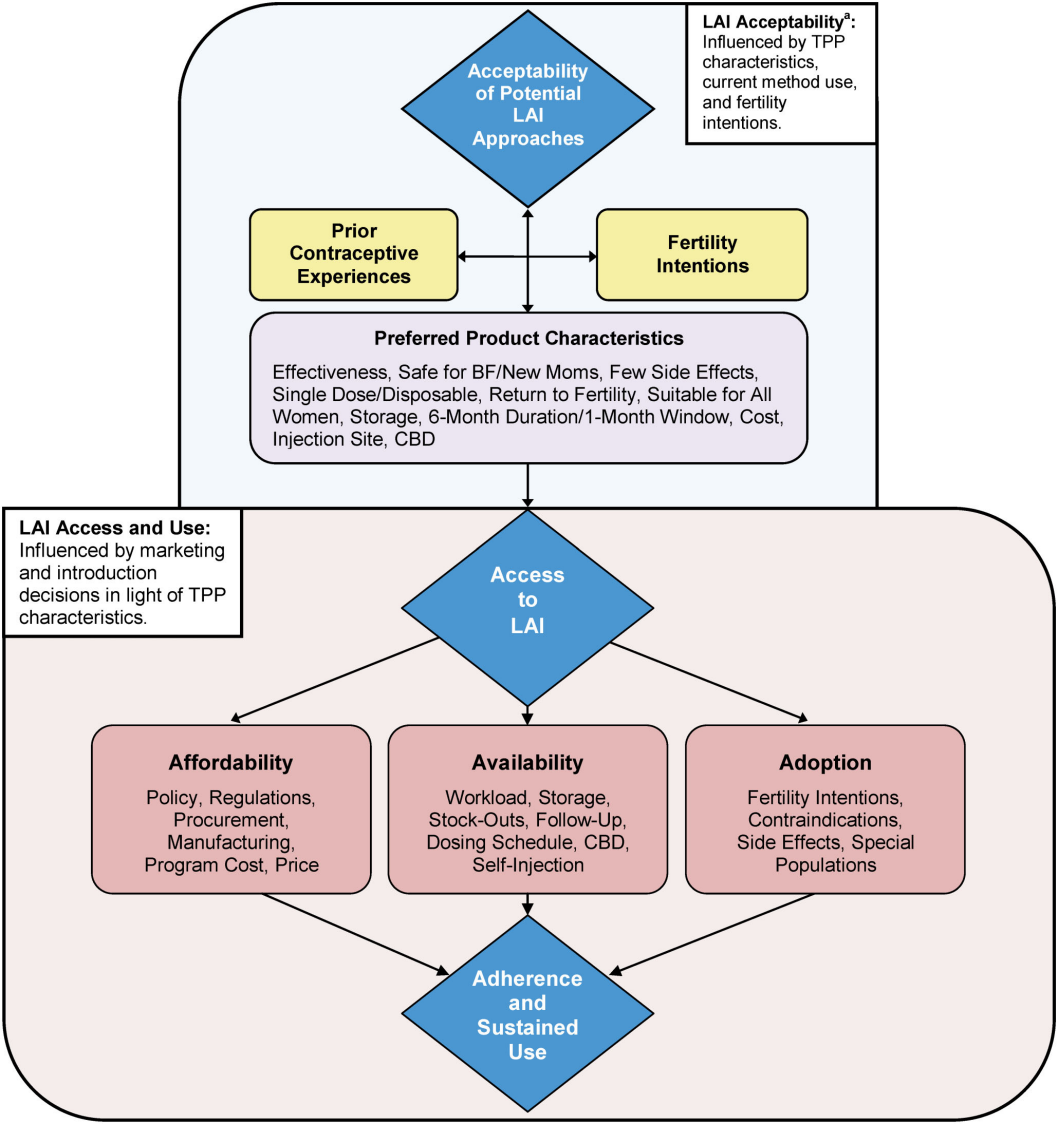
Affordability, Availability, and Adoption: Systems-Level Considerations for Enhancing Access to an LAI Abbreviations: BF, breastfeeding; CBD, community-based distribution; LAI, longer-acting injectable; TPP, target product profile.^a^ For full version of the “Acceptability” portion of this figure, see Tolley et al, 2014.^15^

### Affordability: Policy, Regulatory Bodies, and Manufacturers as Pivotal Components

Delays with regulatory approvals, challenges with procurement, or a lack of partnerships with low-cost manufacturers could hinder cost-effective product introduction and scale-up. These systemic issues could influence pricing, procurement, cost to the health system, and eventual availability of an LAI, which respondents viewed as larger issues than acceptability of the product among clients (Box 2). Although most respondents, especially those at the policy and program levels, believed that an LAI would fit into the national method mix well, procurement price could determine whether a product could be provided through public-sector programs. One question that remains is whether a 6-month injectable contraceptive has the potential to attract new family planning users or whether the current market for injectables will be “cannibalized” by the new LAI, thus resulting in little overall change in contraceptive prevalence rates at the country level. Still, even if a 6-month injectable does not attract high numbers of new family planning users, it is likely that an LAI would improve compliance and continuation rates among those who switch from existing injectables.^18^ Understanding these dynamics better could influence both forecasting for procurement and supply chain management.

**BOX 2. Key Take-Away Messages for Introducing a Longer-Acting Injectable**

**Acceptability.** Service providers, program implementers, and policy makers from Kenya and Rwanda were interested in introducing an LAI into their health care systems and believed that women would find the method acceptable. They also felt that an LAI would fill a gap in the current method mix—especially for women desiring a method with duration somewhere in between that of the long-acting reversible contraceptives (IUDs and implants) and other short-acting methods (including the 3-month injectable).

**Cost.** Cost to health systems is a major consideration in the introduction of a longer-acting injectable. The up-front cost for getting the method approved, training staff on method provision, and establishing distribution processes should be balanced with the likely efficiencies experienced by overburdened health systems. Such efficiencies include a reduction in the number of reinjection visits that providers must deliver as well as the potential for decreased contraceptive discontinuation rates.

**Community-based distribution.** Community-based distribution and other innovative distribution mechanisms for an LAI would have a sizeable impact on enhancing potential users' access to the method.

Cost—to both the health care system and to the end-user—could be lowered by partnering with a manufacturer in an emerging market (eg, Brazil, China, or India), but not without considering the potential trade-off in real or perceived product quality. Other considerations are prioritizing an extended shelf life for the drug, ensuring early policy and program planning to guarantee adequate public-sector budgetary allotment, and fully involving the public, private, and social marketing sectors in a comprehensive introduction strategy to reach potential users.

Creative strategies should be employed by product development groups, donors, governments, and distributors to guarantee access to a low-cost product among target populations in low-resource settings. Effective approaches that have been used with other contraceptives include, but are not limited to, public-sector pricing agreements with distributors, partnerships with manufacturers from emerging markets, and agreements brokered by donors that guarantee funding for set volumes of product in exchange for a lower price per unit.^19^ Additionally, attempts to make registration processes more efficient should be considered. Ongoing early regional harmonization efforts are underway, which may assist in fast-tracking product registration approvals.^20^ Also, local registration partners can help navigate country-specific regulations and advocate product approval.^21^

### Availability: Service Delivery and Distribution Mechanisms

The potential for a shortage of human resources to meet the demand for an LAI is a valid concern that should be examined. Task sharing with CHWs could be a feasible way to enhance service delivery and ensure that clinic-based health workers maintain a manageable client load. Additionally, disseminating updated guidelines on injectable service provision would help address some of the workload concerns among facility-based providers. For example, WHO's 2008 change to the approved reinjection window for the currently available injectable from 2 weeks to 4 weeks should allow more women the flexibility to receive a timely reinjection without the addition of extra appointments—and thus, provider time—to rule out pregnancy before receiving a reinjection.

Allowing CHWs to provide the longer-acting injectable could expand access to the method while also ensuring a manageable workload for clinicians.

To allay provider or client concerns about side effect management given longer periods between doses of the LAI, provider training could be expanded to include proper counseling for clients, and referral systems could be reviewed with providers, or new ones created. Training could build on the similarities to existing injectable products (eg, same mode of administration, contraindications, main counseling issues), while concurrently emphasizing the unique characteristics of this particular method. Ultimately, the introduction of an LAI could also be a timely opportunity to introduce relevant information about the LAI and other injectable methods in order to make providers' jobs less burdensome.

Additionally, policy and program planning for CBD distribution of an LAI, in both the public and private/social marketing sectors, should be addressed early to ensure no delays if and when a new method is introduced. For instance, while this study was being conducted, CBD in Kenya had already been piloted but policy had not yet been changed. If pilot testing shows that access to injectables can be enhanced, policy makers and program managers will want to align national policy and clinical guidelines with usage and distribution for all injectables, including a potential LAI, to increase access for those in need. A particular focus may also be needed on sensitizing mid- and higher-level providers to the benefits of CBD, given that we saw reluctance about CBD from the sample of nurses and midwives in Kenya. Their reluctance may be partially due to fears about losing responsibility and status if CHWs were allowed to provide some of the same services that nurses and midwives currently provide.

Some respondents were supportive about self-injection as a possibility for potential LAI users. Although CBD might be a more viable option initially, alternate channels for service provision such as self-injection should still be considered. When CBD of contraceptives was first introduced, sensitization of all stakeholders and communities and proper training of providers led to many successful CBD programs; the introduction of self-injection of injectable contraceptives could follow a similar path.^13^ Patients with diabetes provide a case in point that people can safely self-inject and manage their fears about injections, which could be emphasized to both providers and users alike. Still, insulin is a very different product, requiring daily injection as opposed to twice-yearly injection of an LAI. Opposition to self-injected contraception might also be based on the misperception that women would need to self-inject with an intramuscular delivery device (instead of a subcutaneous device). Better education about self-injection of contraceptives could potentially alleviate provider reservations.

### Adoption: Drawing on the Preferences of Potential Users

Policy makers, program implementers, providers, and international opinion leaders overwhelmingly had high interest in an LAI and believed that women would as well, particularly if side effects and contraindications were minimized (for the views of potential users themselves, see Tolley et al, 2014^15^). Respondents had clear characterizations of the types of women they saw as potential users—women who already use the 3-month injectable, women who want immediate protection after childbirth and while breastfeeding, spacers in Kenya, and limiters in Rwanda. In some cases, however, these characterizations were partly based on inaccurate or questionable assumptions. These inaccurate characterizations draw attention to the nuances and potential pitfalls of relying on higher-level decision-makers to understand the needs and preferences of a user population. While providers and other stakeholders certainly have a role to play in assessing user population composition and in reaching that population, they are not immune to misperceptions that can hamper women's acceptance of a product. This again highlights the necessity of addressing the introduction of a new method with a holistic approach that considers user perspectives and lived experiences as well as those of providers, program managers, and policy makers. As women's health is a large focus of health systems, and health systems are largely guided by higher-level decisions, programs could enhance their effectiveness by more regularly and systematically “checking the pulse” of users in order to retain a more accurate depiction of user preferences and beliefs.

Also, most respondents from this sample expressed enthusiasm for a product that could be used immediately after birth. However, current WHO guidelines do not recommend initiation of progestin-only methods, including injectables, until 6 weeks postpartum.^22^^,^^23^ If an LAI could be administered after childbirth while women are still at a facility or via a postnatal checkup by a CHW, it would help to ensure that women receive timely and extended protection from rapid repeat pregnancies, therefore increasing healthy birth spacing. Modeling studies comparing possible disadvantages of administering injectables and/or other progestin-only methods immediately following childbirth to potential benefits of reducing morbidity and mortality associated with rapid repeat pregnancies could be potentially useful in steering guidance revisions. This might be a powerful consideration if substantial decreases in unintended pregnancies combined with decreased method discontinuation were resultant from guideline revisions.

### Limitations

This study has several limitations. First, the study had a small sample size; in-depth interviews were conducted in only two countries, and the research used a qualitative design, limiting its generalizability. However, as is true with many qualitative studies, data collection was intended to provide a more in-depth understanding of interest in an LAI, including factors that influence product introduction and potential barriers to access. Second, the sample of opinion leaders was not a representative sample; it was determined via self-selection and therefore some bias is possible. Additionally, as the study was designed to assess the acceptability of key characteristics of a hypothetical LAI, responses collected from participants may have been limited by key informants' lack of knowledge of all facets of product introduction processes. Building on this initial research, future studies that focus on LAI introduction could determine more generalizable estimates for preferred product characteristics or identify most common health systems factors by including a larger sample with expanded country inclusion.

## CONCLUSIONS

Stakeholders in Kenya and Rwanda—two countries with markedly different service delivery environments—were overall enthusiastic about a potential LAI. They thought the method would fill a special niche in their countries' method mix, with the unique attributes of the LAI promoting adoption among potential users both for spacing and for limiting births and to both current injectable users and new family planning clients. Messaging about the method will need to balance similarities of the LAI to other injectables with the LAI's distinctive characteristics. Introduction of a new LAI would be a timely opportunity to offer provider training on the new method as well as refresher training on all types of injectables to ensure providers are aware of and are using the most up-to-date service delivery guidelines. However, use of an LAI by women will largely be dictated by affordability and availability of the method. Creative strategies should be employed to ensure a low-cost product, for example, by partnering with manufacturers from emerging markets, arranging public-sector pricing agreements with distributors, and making registration processes more efficient. In addition, allowing all cadres of health care providers, including CHWs, to administer the product can help ensure wider access to the method.

## Notes

**Peer Reviewed**

**First published online:** 2014 Oct 15
